# High Ammonium Addition Changes the Diversity and Structure of Bacterial Communities in Temperate Wetland Soils of Northeastern China

**DOI:** 10.3390/microorganisms11082033

**Published:** 2023-08-08

**Authors:** Xiaohong Weng, Mingyu Wang, Xin Sui, Beat Frey, Yingnan Liu, Rongtao Zhang, Hongwei Ni, Maihe Li

**Affiliations:** 1 Engineering Research Center of Agricultural Microbiology Technology, Ministry of Education & Heilongjiang Provincial Key Laboratory of Ecological Restoration and Resource Utilization for Cold Region & Key Laboratory of Microbiology, College of Heilongjiang Province & School of Life Sciences, Heilongjiang University, Harbin 150080, China; xiaohongweng@126.com (X.W.); wmy022234@163.com (M.W.); 2 Swiss Federal Institute for Forest, Snow and Landscape Research WSL, 8903 Birmensdorf, Switzerland; beat.frey@wsl.ch; 3 Institute of Nature and Ecology, Heilongjiang Academy of Sciences, Harbin 150001, China; liuyingnan234@163.com (Y.L.); zhangrongtao14@163.com (R.Z.); 4 Heilongjiang Academy of Forestry, Harbin 150022, China; nihongwei2000@163.com; 5 Key Laboratory of Geographical Processes and Ecological Security in Changbai Mountains, Ministry of Education, School of Geographical Sciences, Northeast Normal University, Changchun 130024, China; 6 School of Life Science, Hebei University, Baoding 071002, China

**Keywords:** atmospheric nitrogen addition, bacterial diversity, community structure, network, *Deyeuxia angustifolia* wetland

## Abstract

The soil microbiome is an important component of wetland ecosystems and plays a pivotal role in nutrient cycling and climate regulation. Nitrogen (N) addition influences the soil’s microbial diversity, composition, and function by affecting the soil’s nutrient status. The change in soil bacterial diversity and composition in temperate wetland ecosystems in response to high ammonium nitrogen additions remains unclear. In this study, we used high-throughput sequencing technology to study the changes of soil bacterial diversity and community structure with increasing ammonium concentrations [CK (control, 0 kg ha^−1^ a^−1^), LN (low nitrogen addition, 40 kg ha^−1^ a^−1^), and HN (high nitrogen addition, 80 kg ha^−1^ a^−1^)] at a field experimental site in the Sanjiang Plain wetland, China. Our results showed that except for soil organic carbon (SOC), other soil physicochemical parameters, i.e., soil moisture content (SMC), dissolved organic nitrogen (DON), total nitrogen (TN), pH, ammonium nitrogen (NH_4_^+^), and dissolved organic carbon (DOC), changed significantly among three ammonium nitrogen addition concentrations (*p* < 0.05). Compared to CK, LN did not change soil bacterial α-diversity (*p* > 0.05), and HN only decreased the Shannon (*p* < 0.05) and did not change the Chao (*p* > 0.05) indices of soil bacterial community. Ammonium nitrogen addition did not significantly affect the soil’s bacterial community structure based on non-metric multidimensional scaling (NMDS) and PERMANOVA (ADONIS) analyses. Acidobacteriota (24.96–31.11%), Proteobacteria (16.82–26.78%), Chloroflexi (10.34–18.09%), Verrucomicrobiota (5.23–11.56%), and Actinobacteriota (5.63–8.75%) were the most abundant bacterial phyla in the soils. Nitrogen addition changed the complexity and stability of the bacterial network. SMC, NO_3_^−^, and pH were the main drivers of the bacterial community structure. These findings indicate that enhanced atmospheric nitrogen addition may have an impact on bacterial communities in soil, and this study will allow us to better understand the response of the soil microbiome in wetland ecosystems in the framework of increasing nitrogen deposition.

## 1. Introduction

In recent decades, agricultural fertilization, combustion of fossil fuels, and other human activities have increased nitrogen inputs to wetland ecosystems [1]. Therefore, external nitrogen addition and atmospheric nitrogen deposition can directly or indirectly affect soil’s aboveground biological processes and underground biochemistry [2,3]. Nitrogen addition can influence soil’s microbial communities by alleviating resource limitations [4] and altering its physiochemical properties [5]. Conversely, as an important part of regulating the underground ecological process, the dynamic changes in soil microorganisms can also provide feedback to ecosystem functions [6] by affecting litter decomposition [7,8], nutrient cycling, and greenhouse gas emissions [9]. Nevertheless, despite the critical contributions of soil microorganisms to the underground ecological process and ecosystem stability, the studies that characterized the effects of nitrogen addition on soil microbial community structure and diversity were still limited.

Soil bacteria are a major component of soil microorganisms and play crucial functions in the ecosystem [10,11]. Moreover, their diversity, composition, complexity, and stability are particularly sensitive to changes in nitrogen content [12]. Several studies have shown that nitrogen addition can lead to major shifts in various edaphic parameters, which affect soil bacterial communities. For example, N addition indirectly affected the bacterial community composition by decreasing soil pH [3], and Song et al. [8] found that increased N availability enhanced bacterial activity. Li et al. [13] and Xie et al. [14] found that increasing N input significantly affected the soil’s microbial species interactions and stability, which in turn affects the stability of the microbial community structure [13,15]. Moreover, soil N availability also played an important role in shaping microbial networks [16], such as ammonium nitrogen (NH_4_^+^-N) concentration enhanced inter-microbial interactions [17]. Some studies indicated that nitrogen addition significantly changed the relative abundance of bacteria [18,19]. For example, nitrogen addition enhanced the relative abundance of Chloroflexi, and reduced the relative abundance of Chlorobi and Verrucomicrobia in a coastal wetland [20]. Zhang et al. [21] also found that N addition increased the relative abundance of Chloroflexi but decreased the relative abundance of Firmicutes and Bacteroidetes in *Deyeuxia angustifolia* wetland. In contrast, Hu et al. [2] reported that high nitrogen treatment significantly reduced the relative abundance of Chloroflexi in alpine wetland. The varied results may be attributed to the different forms of nitrogen application because the above studies usually used urea or ammonium nitrate fertilizer (NH_4_NO_3_) to simulate the nitrogen deposition. However, the responses of bacterial communities to ammonium nitrogen (NH_4_^+^) inputs remain lacking in studies.

Ammonium nitrogen is the dominant form of atmospheric nitrogen deposition [22]. Ammonium nitrogen may increase aboveground biomass [23], stimulate soil bacterial activities [24], increase the ratio of NH_4_^+^ to NO_3_^−^, and affect the rate of soil N transformation [25]. In addition, ammonium nitrogen can produce more protons than nitrate nitrogen in the soil [26], and bacteria preferentially assimilate NH_4_^+^ over NO_3_^−^ due to the lower energy expenditure for NH_4_^+^ assimilation [27]. At present, many studies have been conducted on the effects of ammonium nitrogen addition to soil bacteria. For example, Zhang et al. [28] evaluated the biomass of soil microbial communities responding to ammonium (NH_4_Cl) application in a slash pine (*Pinus elliottii*) plantation in a subtropical forest ecosystem, and found that ammonium treatments changed the bacterial content of phospholipid fatty acids (PLFAs). Liu et al. [29] examined soil bacteria in Chinese fir plantations using plate counts and PLFA analysis and found that the addition of ammonium (NH_4_^+^) increased bacterial abundance and biomass. Geng et al. [30] found that ammonium addition changed bacterial community structure and increased the ratio of fungi/bacteria (F/B) in a boreal forest. However, these studies were mainly carried out in forest ecosystems, and the response of bacteria to ammonium nitrogen in wetland ecosystems remains unclear.

Sanjiang Plain is a typical wetland, representative of the global temperate wetland ecosystem [31]. This freshwater marsh wetland is the largest concentrated distribution in the northeast of China and is one of the most sensitive areas to global changes [32]. *D. angutifolia* wetland is the main wetland type in the Sanjiang Plain, accounting for 34.45% of Sanjiang Plain wetland [33]. The nitrogen deposition concentrations of *D. angutifolia* wetland have increased severely, and this is attributed to agricultural and industrial activities [34,35]. These activities’ ecological impacts on soil microbial communities have been of great concern in recent years. For example, Song et al. [8] found that N addition increased litter decomposition rates through enhanced microbial activity. Zhang et al. [21] showed that N addition changed the structure and abundance of bacterial communities. Sui et al. [36] indicated that N addition reduced the abundance of some major bacterial groups, but that there was no significant impact on the bacterial community structure. The above studies mainly used NH_4_NO_3_ as a nitrogen source, however, the NH_4_^+^ form was one of the primary forms of nitrogen deposition in the Sanjiang Plain [34] and, to our knowledge, there are no studies on the response of the bacterial community to NH_4_^+^ addition in Sanjiang Plain. Therefore, our study investigated the changes in the soil bacterial community, diversity, and stability using high-throughput sequencing technology and the correlation with the soil’s physicochemical parameters in response to NH_4_^+^ addition on the experimental platform of the Sanjiang Plain. We propose the following hypotheses: (1) NH_4_^+^ addition would change the soil’s bacterial structure, changing the complexity and stability of the network structure of the bacterial community. (2) High nitrogen would reduce bacterial diversity more strongly than low nitrogen. This research will be of great significance for comprehensively evaluating wetland nitrogen cycling and improving sustainable wetland management in Northeastern China.

## 2. Materials and Methods

### 2.1. Study Area

This study area is located in the Honghe National Nature Reserve of Sanjiang Plain (47°42′18″–47°52′07″ N, 133°34′38″–133°46′29″ E) (Supplementary Figure S1). The annual average temperature and evaporation are 1.9 °C and 1166 mm, respectively. The annual precipitation is 585 mm, mostly concentrated from July to September. Northwest winds prevail throughout the year, and the ≥10 °C effective accumulated temperature is 2165~2624 °C, with an annual frost-free period of 131 day. The soil belongs to the categories of bleached stagnant soil and fibrous organic soil [37]. The main wetland types are meadow wetland, meadow swamp wetland, and swamp wetland. The dominant vegetations are *D. angustifolia*, *Glyceria spiculose*, *Carex lasiocarpa*, and *Carex pseudocuraica*.

### 2.2. Soil Sampling

The field experiment was established in May 2016 with three concentration levels: control (CK: 0 kg NH_4_^+^ ha^−1^ year), low nitrogen (LN: 40 kg NH_4_^+^ ha^−1^ year), and high nitrogen (HN: 80 kg NH_4_^+^ ha^−1^ year). The low nitrogen addition concentrations (40 kg N ha^−1^ year) were set up based on the local fertilizer application and the current status of atmospheric N deposition in China [21]. The high nitrogen addition concentrations (80 kg N ha^−1^ year) were set up to predict the impact of continued N input on soil microorganisms in the future. Each treatment had three replicates (9 plots in total) and each plot was 10 m × 10 m in area. NH_4_Cl was used as the N source, dissolved in water, and sprayed uniformly in May each year, and CK was sprayed with an equal amount of water. In October 2021 (five and half years after the beginning of the experiment), 15 to 20 soil samples (0~20 cm) were randomly obtained using a soil auger (5 cm in diameter, 20 cm deep) after removing the litter layer from each plot. The soil samples were mixed into one soil sample to represent the soil sample of the plot, after removing impurities such as vegetation fallout from the sample, and then placed in a carrying incubator at 4 °C and quickly transported to the laboratory. The soil samples were divided into two parts. One was stored at −80 °C for the determination of the soil’s bacterial community, and another was air-dried, thoroughly ground, and sieved by 2 mm for the determination of its physicochemical properties.

### 2.3. Determination of Soil Physicochemical Properties

Soil moisture content (SMC) was determined via the drying method [33]; soil pH was measured by a water–soil ratio of 1:2.5; soil total nitrogen (TN) was measured by the Autoanalyzer (AutoAnalyzer 3, Hamburg, Germany); soil organic carbon (SOC) was determined by an elemental analyzer (VarioEL III, Frankfurt, Germany); nitrate nitrogen (NO_3_^−^) and ammonium nitrogen (NH_4_^+^) were measured by the Autoanalyzer (AutoAnalyzer 3, Hamburg, Germany); dissolved organic carbon (DOC) and dissolved organic nitrogen (DON) were determined by a K_2_SO_4_ leaching method at a ratio of 1:5 soil to liquid, extracted for 30 min, centrifuged and filtered, and then measured by a TOC analyzer [38].

### 2.4. DNA Extraction

Soil bacterial DNA was extracted using the E.Z.N.A.^®^ Soil DNA Kit (Omega Bio-tek, Norcross, GA, USA). PCR amplification was performed with the primer pairs 515F (5′-barcode-GTGCCAGCMGCCGCGG-3′) and 907R (5′-CCGTCAATTCMTTTRAGTTT-3′) spanning the variable region V4-V5 of the 16S rRNA gene [39], where the barcode is an eight-base sequence unique to each sample. PCR reactions were made in a total volume of 20 μL that contained 4 μL of 5 × FastPfu Buffer, 2 μL of 2.5 mM dNTPs, 0.8 μL of each primer (5 μM), 0.4 μL of FastPfu Polymerase, 10 ng of template DNA, and finally ddH_2_O up to 20 μL. PCR amplification conditions were as follows: 95 °C for 2 min, followed by 25 cycles of 95 °C for 30 s, 55 °C for 30 s, 72 °C for 30 s, and a final extension at 72 °C for 5 min. Three replicates of each sample were made and PCR products from the same sample were pooled and subjected to agarose electrophoresis and purified using the AxyPrep DNA Gel Extraction Kit (Axygen Biosciences, Union City, CA, USA). Purified PCR products were quantified using Qubit^®^ 3.0 (Life Invitrogen, Waltham, USA) and mixed in the appropriate proportions as required for the sequencing volume of each sample. Sequencing was performed on an Illumina MiSeq platform (Shanghai BIOZERON Co., Ltd., Shanghai, China). All raw sequences have been deposited into an NCBI Sequence Read Archive with the BioProject accession: PRJNA880534.

### 2.5. Bioinformatics

Raw bacterial fastq files were de-multiplexed, quality filtered, and assessed through QIIME 2 (version 2022.2). Forward and reverse reads were merged using PEAR (Paired-End reAd mergeR, version 0.9.6) software. Low quality sequences less than 200 bp in length and sequences with an average quality score less than 20 were removed before further analysis. Exact barcode matching was implemented, allowing for a two-nucleotide mismatch in the primer matching. Reads including unclear characters were also included for removal. The trimmed sequences were checked for chimera, and those with chimeras were removed using the Uchime algorithm. Only sequences with an overlap of more than 10 bp were assembled based on their overlapping sequences; reads that were not able to be assembled were discarded. Sequences were clustered into operational taxonomic units (OTUs) at 97% similarity using UPARSE. With respect to taxonomy, the obtained OTUs were annotated using the SILVA database (version. 138). We rarefied the data set to the lowest number of sequences for all the samples and used this data set for further community analysis.

### 2.6. Statistical Analyses

One-way ANOVA and a Duncan test were used to analyze the effect of different levels of N addition on the soil’s physicochemical properties, and *p* < 0.05 was considered statistically significant via using the SPSS 20.0 (SPSS, Inc., Chicago, IL, USA). The rarefaction curve was created using R (version 4.4.3) software. Stamp analysis [40] was used to produce box plots of diversity indices and to analyze differences in bacterial community composition (phylum and genus level) between the different nitrogen addition treatments. A correlation matrix was constructed to look for relationships between soil properties and bacterial community by using the “Hmisc” package of R. The α diversity indices (Chao1, Shannon, and Simpson) were performed by Mothur (version 1.30.1). Redundancy analysis (RDA) and non-metric multidimensional scaling (NMDS) (using Bray–Curtis dissimilarity) and a Venn diagram were achieved using the vegan package in the R program (version 4.1.0). Indicator species analysis of soil bacterial composition under different N treatments were analyzed using the LEfSe component of the software Galaxy 21.09 (LDA = 3.5). For the network analysis, only OTUs with >1% abundance were selected and imported into the microplatform (http://www.cloud.biomicroclass.com/CloudPlatform/SoftPage/IGC) for intra-group correlation analysis (Pearson correlation) (accessed on 26 March 2023). Data files for edges and nodes were downloaded, and edge data with *p* < 0.05 were selected to import into Cytoscape for network plotting.

## 3. Result

### 3.1. Changes of Soil Physicochemical Characteristics under Different NH_4_^+^ Addition

Different nitrogen addition treatments had different effects on the soil’s physicochemical parameters (Table 1). The SMC, DON, and TN significantly increased (*p* < 0.05) and, inversely, pH significantly decreased (*p* < 0.05) with increasing concentrations of N addition. Compared with CK, HN treatment increased the contents of NO_3_^−^ and DOC, whereas it decreased the NH_4_^+^, while LN treatment decreased the NH_4_^+^ and DOC (*p* < 0.05).

### 3.2. Sequencing Data Analysis and Distribution of Soil Bacterial Community

After high-throughput sequencing and optimization, a total of 429,316 sequences were obtained from the amplicons. A total of 178,818,450 bp was obtained, with a mean read length of 416.59 bp. The reads of 401–450 bp comprised 99.56% of the total sequences obtained and rarefaction curves were close to saturation, indicating that the sequencing data were representative of the real situation of the wetland soil bacterial community (Supplementary Figure S2).

In total, 7727 OTUs were detected, of which 3247 (42.02%) were shared between the CK, LN, and HN treatments (Supplementary Figure S3). CK samples produced the highest number of specific OTUs (912, accounting for 11.80%), while 743 OTUs were specific to LN and 675 OTUs were specific to HN, accounting for 9.62% and 8.74%, respectively. A large proportion of OTUs were shared in CK and LN (16.08%), while 5.99% of OTUs were shared between LN and HN, and 5.75% were isolated from both CK and HN. Therefore, the distribution of soil bacteria OTUs in the ecological network was significantly influenced by the nitrogen addition in the *D. angustifolia* wetlands of the Sanjiang Plain.

### 3.3. Increasing Ammonium Addition Influenced Bacterial α-Diversity

**Figure 1:**
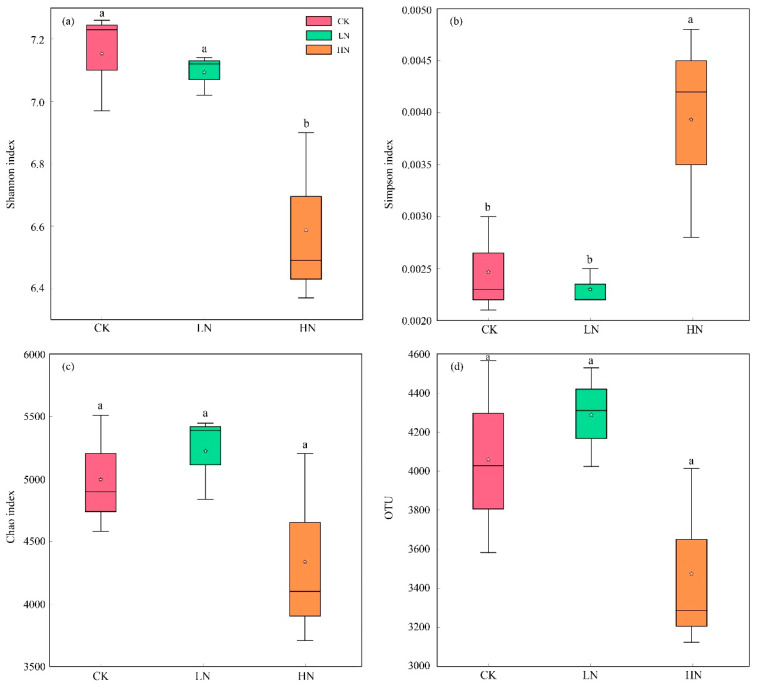
Effects of N application on soil microbial community α-diversity. (**a**) Shannon index analysis of bacteria. (**b**) Simpson index analysis of bacteria. (**c**) Chao index analysis of bacteria. (**d**) OTU numbers of bacteria. Lowercase letters indicate significant differences (*p* < 0.05). The asterisk indicates the average of each group. CK: control; LN: low ammonium; HN: high ammonium.

The α-diversities of the three nitrogen addition treatments are shown in Figure 1. Compared with CK, LN did not significantly change the Shannon index and the Simpson index (*p* > 0.05), but HN significantly decreased the Shannon index and increased the Simpson index (*p* < 0.05). Thus, the HN treatment significantly reduced the heterogeneity of the soil bacterial community. The Chao1 index and OTU numbers did not significantly differ (*p* > 0.05) between the CK, LN, and HN treatment, indicating that N addition did not change the richness of the soil bacterial community.

**Figure 2:**
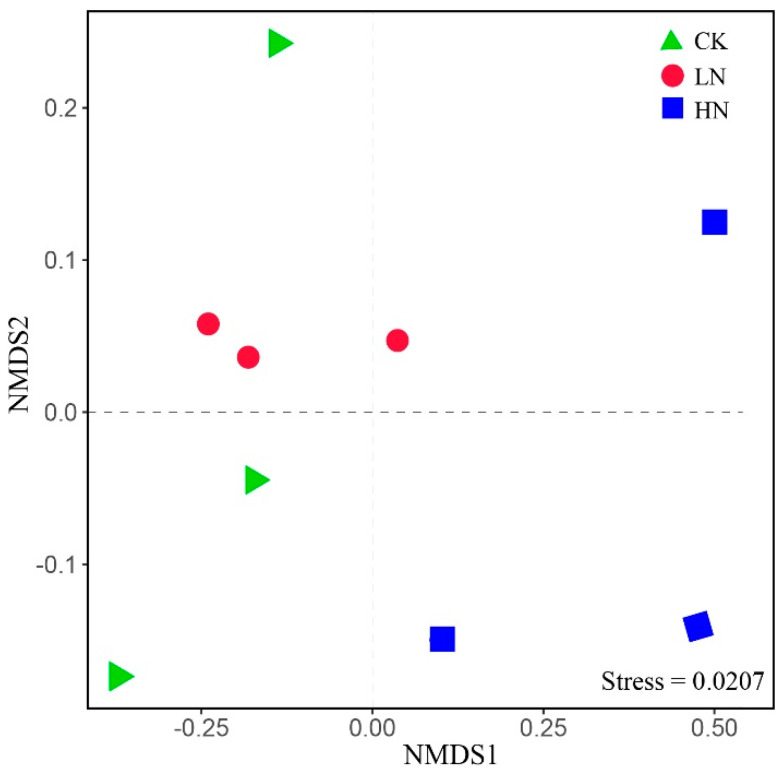
Non-metric multidimensional scaling (NMDS) of bacterial communities under different N addition levels. CK: control; LN: low ammonium; HN: high ammonium.

The effect of ammonium addition on bacterial community composition was demonstrated by non-metric multidimensional scaling (NMDS) based on Bray–Curtis dissimilarity (Figure 2). The soil bacterial community structure of LN, HN, and CK were significantly separated (Table S1, R^2^ = 0.48 and *p* < 0.05), indicating that the higher ammonium addition significantly affected the bacterial community structure. Interestingly, the PERMANOVA analysis showed that soil bacterial community structure of LN and HN did not significantly change compared to CK (Table S1).

### 3.4. Soil Bacterial Community Composition Was Changed by Ammonium Addition

**Figure 3:**
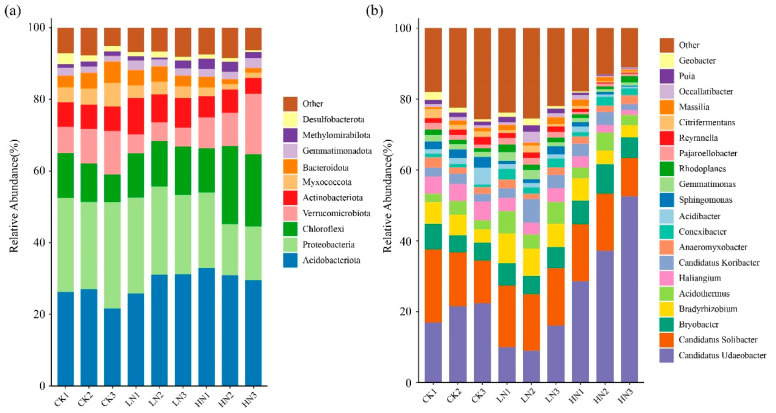
Relative abundance of bacterial phyla. (**a**) Top 10 phyla and genera, (**b**) top 20 genera under different N addition levels. “Other” of the legend in (**a**) includes 34 phyla except top 10 bacterial phyla. “Other” of the legend in (**b**) includes 53 genera except after top 20 bacterial genera. CK: control; LN: low ammonium; HN: high ammonium.

A total of 7727 bacterial OTUs belonged to 44 phyla. The Acidobacteriota (24.96–31.11%), Proteobacteria (16.82–26.78%), Chloroflexi (10.34–18.09%), Verrucomicrobiota (5.23–11.56%), and Actinobacteriota (5.63–8.75%) were the most abundant bacterial phyla across all N treatments, and they represented approximately 80.85% of the reads from bacterial phyla (Figure 3). LN did not significantly change bacterial phyla (*p* > 0.05). HN treatment significantly increased the relative abundance of Acidobacteriota, but significantly decreased the relative abundance of Proteobacteria and Myxococcota (Supplementary Figure S5, *p* < 0.05).

At the genus level, the dominated bacterial genera were *Candidatus Udaeobacter* (11.66–39.45%), *Candidatus Solibacter* (14.32–16.51%), *Bryobacter* (5.66–6.92%), *Bradyrhizobium* (4.48–7.54%), *Acidothermus* (2.99–5.55%), *Haliangium* (2.13–4.91%), *Candidatus Koribacter* (2.55–4.44%), and *Anaeromyxobacter* (1.91–2.10%), and they represented approximately 64.28% of the reads from bacterial genera (Figure 3). Furthermore, compared with CK, the LN treatment significantly increased the relative abundances of *Bradyrhizobium*, *Acidothermus*, and *Conexibacter* and decreased the relative abundances of *Haliangium* (Supplementary Figure S6, *p* < 0.05). HN treatment significantly decreased the relative abundances of *Gemmatimonas*, *Sphingomonas*, *Haliangium*, *Puia*, *Reyanella*, and *Citrifermentans* (Supplementary Figure S7, *p* < 0.05).

### 3.5. Indicator Analysis of Soil Bacterial Community Responding to Ammonium Addition

**Figure 4:**
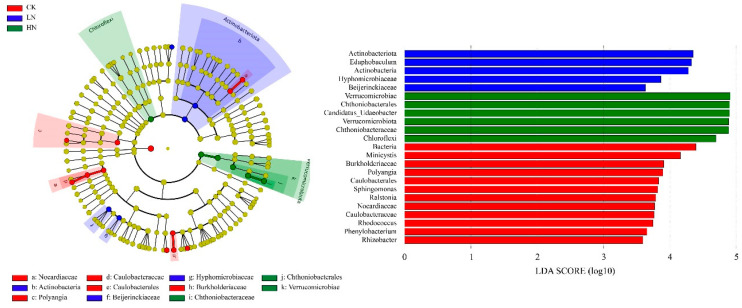
The cladogram of the soil bacteria community under different N addition levels (LDA score = 3.5). CK: control; LN: low ammonium; HN: high ammonium.

The cladogram of the soil bacterial community under different N addition treatments with an LDA score of 3.5 and 23 taxa exhibited significant differences among the three N treatments (Figure 4). Each node in the graph represents a bacterial taxon, with non-significant bacteria labelled using yellow nodes and significant bacteria given other colors (red, blue, and green). The colors represent the subgroups in which they were abundant, i.e., they indicate that these branches had a higher abundance in that subgroup. As seen in the Figure 4, *Minicystis* of Myxococcota, *Rhodococcus* of Actinobacterita, *Sphingomonas*, *Ralstonia*, *Phenylobacterium*, and *Rhizobacter* of Proteobacteria were indicator genera in CK; *Edaphobaculum* was an indicator genus in LN; and *Candidatus_Udaeobacter* was an indicator genus in HN.

### 3.6. Ammonium Addition Changed the Bacterial Network Structure and Complexity

**Figure 5:**
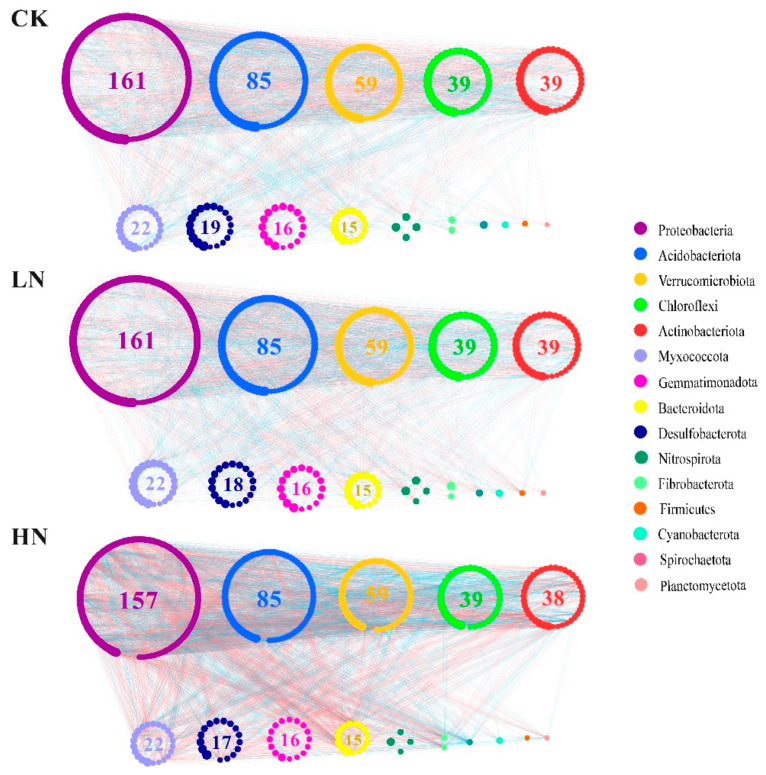
Network structures of bacteria under different N addition levels. Each node signifies an OTU. The size of the node represents the degree of node. Colors of the nodes indicate different phyla. The values in the circle represent the OTU numbers of the corresponding phylum. Colors of the lines represent the strength of the correlation between OTUs. A red line indicates a negative interaction between two individual nodes, while a blue line indicates a negative interaction. CK: control; LN: low ammonium; HN: high ammonium.

A network can be developed to represent the interaction and connectivity among different bacterial populations carrying the OTUs (Figure 5). The nodes of all the networks were assigned to 15 bacterial phyla. The OTU numbers, average geodesic distance, and number of modules under LN and HN were smaller than those of CK, but the average clustering coefficient increased from CK to HN (Table 2), indicating that LN and HN reduced the stability of the bacterial network. Total links (positive and negative links), average degree, and network density all showed a tendency as HN > CK > LN, indicating that HN increased the complexity of the bacterial network, whereas LN decreased the complexity compared to CK. These results indicated that N addition changed the complexity and stability of the bacterial network.

### 3.7. Relationships between Microbial Communities and Soil Properties

**Figure 6:**
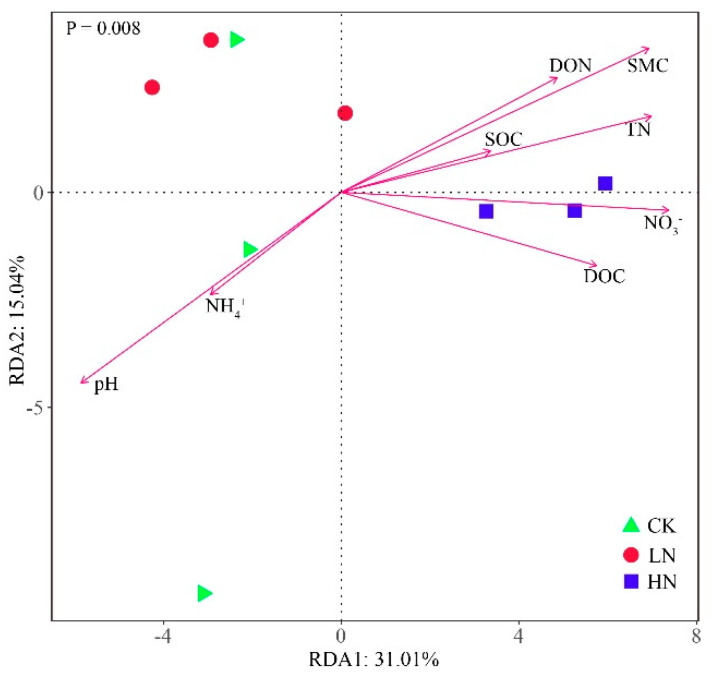
Redundancy analysis (RDA) of the relationships between soil properties and bacterial community structure under N different addition. CK: control; LN: low ammonium; HN: high ammonium.

The effects of soil properties on bacterial communities were analyzed using RDA (Figure 6). SMC, pH, and NO_3_^−^ were the three most influential factors contributing to the changes in bacterial communities (Table S3), with the first two RDA axes accounting for 46.05% of the variation in bacterial communities. The soil bacterial communities under both CK and LN treatments were positively correlated with NH_4_^+^ and pH, whereas they were inhibited by NO_3_^−^, SMC, DOC, TN, DON, and SOC. The bacterial community under HN treatment showed positive correlations with NO_3_^−^, SMC, DOC, TN, DON, and SOC, and negative correlations with the soil pH and NH_4_^+^.

**Figure 7:**
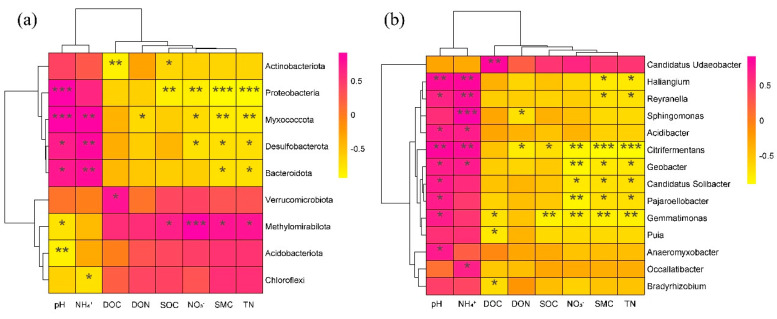
Pearson correlations of bacterial abundant phyla (**a**), and genera (**b**), with soil properties. * indicates 0.5 level; ** indicates 0.01 level; *** indicates 0.001 level. The color gradient (pink to yellow) represents the correlation degree from higher to lower. Pink represents positive correlation and yellow represents negative correlation. CK: control; LN: low ammonium; HN: high ammonium.

The relative abundance of the top 10 phyla and top 20 genera of soil bacteria were correlated with environmental factors, and the results showed that almost all phyla and 14 genera were significantly correlated with environmental factors (Figure 7). At the phylum level (Figure 7a), the abundances of Acidobacteriota, Proteobacteriota, Myxococcota, Desulfobacterota, and Bacteroidota presented a positive relationship with pH and NH_4_^+^, while they showed a negative relationship with DOC, DON, SOC, NO_3_^−^, SMC, and TN. Verrucomicrobiota, Methylomirabilota, Acidobacteriota, and Chloroflexi showed the opposite results. At the genus level (Figure 7b), 13 genera positively correlated with pH and NH_4_^+^, while they showed a negative relationship with DOC, DON, SOC, and NO_3_^−^. In contrast, *Candidatus_Udaeobacter* showed a positive relationship with DOC, DON, SOC, and NO_3_^−^.

## 4. Discussion

### 4.1. Changes in Soil Bacterial Community Diversity and Structure

In accordance with our second hypothesis, our results showed that HN decreased the Shannon diversity and community richness, but LN had no significant impact on the bacterial α diversity (Figure 1). Nitrogen input to soil may change the nitrogen’s ecological niche occupied by different species in the ecosystem [41], which in turn may cause changes in bacterial α diversity [42]. The difference between the Shannon index and the Simpson index in HN was related to species-relative abundance and species richness. The Simpson index was particularly sensitive to the abundance of dominant species, and HN significantly reduced the relative abundance of *Gemmatimonas*, *Sphingomonas*, *Haliangium*, *Puia*, *Reyranella*, and *Citrifermentans* (Figure S7), which may have caused an increase in the Simpson index. As for the Shannon index, which is closely related to the complexity of the communities, the reduction in species richness under HN would reduce the Shannon index (Figure 1 and Figure S3). N addition may increase N availability, changing the substrate utilization patterns in bacterial communities [43], reducing the utilization of carbon sources by the bacterial community, and decreasing the metabolic activity and diversity of the bacteria [29]. Our results were consistent with the findings of previous studies, which showed that increasing N addition concentrations reduced bacterial community diversity [18,44]. High nitrogen addition reduced soil pH, causing acidification of the soil, and therefore changing the diversity of the soil bacterial community [45]. However, previous studies are inconsistent with our findings [2,46]. For example, Hu et al. [2] conducted research on soil bacterial community in a swamp meadow wetland and found that short-term nitrogen application has no significant effect on the microbial diversity. This may be due to the duration of nitrogen addition. In our study, the 5-year N addition may have alleviated the limitation of nitrogen use by the soil bacterial community and caused a saturated state of N in the soil, therefore affecting the soil bacterial community. In addition, the differences in plant types on the ground may have caused differences in plant root exudates and ground litter, resulting in different soil environments, which in turn have different effects on the soil bacterial community [47,48].

According to the NMDS analysis, NH_4_^+^ addition changed the bacterial community structure (Figure 2, Supplementary Table S1), which is in accordance with our first hypothesis. The CK and LN were located in the negative half-axis of NMDS1, and HN was in the positive half-axis of NMDS1, indicating that HN had a greater effect on the soil bacterial community structure than LN. The reason may be that the continuous high NH_4_^+^ input altered the soil environment more than the low NH_4_^+^ concentration, and RDA analysis showed that SMC, NO_3_^−^, and pH were the main factors affecting the bacterial community structure, which was sensitive to NH_4_^+^ concentration. Therefore, the continuous input of N changed the soil’s environmental factors and affected the bacterial community structure [2,15]. However, Sha et al. [49] investigated six years of nitrogen addition on soil microorganisms in a desert ecosystem (NH_4_NO_3_ as nitrogen source) and found that enhanced atmospheric nitrogen addition did not change the bacterial community structure. The differences with our results may be due to the inconsistency of the nitrogen sources applied in the study. Different forms of N input may have different effects on soil bacteria owing to preferential uptake by plants and competition between plant and microbial communities [23,50]. Furthermore, bacteria have different ways of using NH_4_^+^ and NO_3_^−^; NH_4_^+^ can be directly used by bacteria, while bacteria need to reduce NO_3_^−^ to NH_4_^+^ before assimilating it [50,51,52,53]. In addition, the wetland is formed by land–water interaction, and the wetland ecosystem is more vulnerable and sensitive to environmental changes when compared with the desert ecosystem [2]. Therefore, the response of wetland ecosystems to nitrogen inputs would be more significant.

### 4.2. Effects of N Addition on the Soil Bacterial Community Composition

Nitrogen addition significantly changed the composition of the soil’s bacterial phyla communities (*p* < 0.05) (Figure 3, Supplementary Figures S4–S7). In our study, we found that Acidobacteriota, Proteobacteria, and Chloroflexi were essential components of the soil bacteria occurring in the temperate wetland, which is consistent with those found in other wetland ecosystems [2,54]. According to oligotrophic–copiotrophic theory, fast-growing copiotrophic taxa are more likely to grow under nutrient-rich conditions. Conversely, oligotrophic taxa can survive under lower nutrient conditions, since they have a low growth rate [55]. Acidobacteria and Chloroflexi are regarded as oligotrophic microorganisms, while Proteobacteria are considered to be copiotrophic microorganisms [55,56]. However, in our study, we observed that nitrogen addition significantly increased the relative abundance of Acidobacteriota and Chloroflexi, whereas it decreased the Proteobacteria (*p* < 0.05) (Supplementary Figures S4 and S5). However, these results were contrary to the oligotrophic–copiotrophic theory results [57,58]. The reason for this result could possibly be explained by the changes in the soil’s physicochemical properties resulting from the continued ammonium nitrogen addition. The Acidobacteriota is generally acidophilic bacteria [56], and Acidobacteria is usually negatively correlated with soil pH (Figure 7; [59,60]). Therefore, ammonium nitrogen addition may be increasing the relative abundance of Acidobacteriota by reducing soil pH [45,61]. Soil bacterial communities have a narrow pH range for optimal growth, and acidification makes the environment more restrictive, which may also be a reason for the change in the relative abundance of the Proteobacteria [28,62]. Low pH would lead to a decrease in the relative abundance of Proteobacteria, which positively correlated with soil pH [63,64]. According to previous studies, the Chloroflexi were highly tolerant to eutrophication, and they contributed to nutrient removal [65,66]; nitrogen addition increased the nitrogen availability, which may significantly increase the relative abundance of Chloroflexi. Furthermore, the RDA showed that SMC was the most influential factor, resulting in changes in the bacterial community (Figure 6, Supplementary Table S3), and bacteria are sensitive to the moisture content of the soil (Figure 6, [67,68]). The changes in SMC caused by ammonium nitrogen addition may have an impact on the growth and metabolic activities of bacteria, causing changes in the composition of the soil bacterial phyla [69,70,71].

At the genus level, *Candidatus Udaeobacter*, *Candidatus Solibacter*, *Bryobacter*, *Bradyrhizobium*, and *Acidothermus* were the main bacterial genera in all soil samples. Nitrogen addition significantly altered the relative abundance of *Bradyrhizobium* and *Acidothermus* (Figure 3, Supplementary Figure S5). These are important bacterial communities participating in the carbon and nitrogen cycles in soils [72,73,74]. In our study, LN treatment significantly increased the relative abundance of *Bradyrhizobium* and *Acidothermus*. These changes can be explained by the co-trophic hypothesis. *Bradyrhizobium* and *Acidothermus* are classified as copiotrophs [56], and nitrogen addition enriched the level of available N in the soil and therefore stimulated their growth [75]. Xu et al. [76] found that nitrogen addition increased the relative abundance of *Bradyrhizobium*, which is consistent with our results. *Bradyrhizobium* is a parthenogenic nitrogen-fixing microorganism involved in the nitrate and nitrite reduction pathways [77,78]. The process of biological nitrogen fixation requires large amounts of energy and is mainly dependent on soil nutrients, which are increased by the addition of nitrogen, thus increasing the abundance of the nitrogen-fixing bacteria *Bradyrhizobium*. However, Wan et al. (2021) found that nitrogen addition decreased the relative abundance of *Bradyrhizobium*. The differences may be explained by differences in the concentration of nitrogen applied. In our study, LN significantly increased the relative abundance of *Bradyrhizobium*, but HN showed inhibition, although not significantly (Supplementary Figure S7). The implication is that there is a degree of concentration between LN and HN, which when exceeded will inhibit the growth of *Bradyrhizobium* [79]. Furthermore, the pH in the soil may have a strong influence on *Acidothermus*, which are acidophilic genus [73]. The reduction in soil pH provided a suitable environment for the growth of *Acidothermus* (Table 1), increasing the relative abundance of *Acidothermus* [80]. However, Wan et al. [81] found that N addition reduced the relative abundance of Acidothermus in orchard ecosystems, and previous studies found that *Acidothermus* was positively correlated with soil organic matter content [73], which is contrary to our findings. This may be explained by differences in the ecosystem types and the geographical regions and vegetations of different studies. This study was in a temperate natural wetland and the aboveground vegetations were dominated by *D. angustifolia*, so the difference may be attributed to differences in land use patterns and species of plants on the aboveground, compared to previous studies. Moreover, bacterial community composition is not only influenced by the physical and chemical properties of the soil, but also by altitude [82] and temperature and rainfall [83], and the dominating factors may be different in different regions or at different scales.

### 4.3. Changes in the Structure of Soil Bacterial Networks

Analysis of ecological network structure can reveal the relationship between the complexity and stability of ecosystems [84]. In accordance with our first hypothesis, our study showed that nitrogen addition changed potential bacterial competition and network complexity (Figure 5, Table 2). Compared with CK, LN and HN increased the average clustering coefficient of the network but decreased the average geodesic distance of the network. This implied that the application of NH_4_^+^ might remit the competition within bacteria. The nitrogen addition enhanced the efficiency of inter-species transport, which rapidly distributed the effects of disturbance across the network and contributed to a reduction in network stability [85,86]. NH_4_^+^ input changed the total links and average degree of the bacterial network, indicating that nitrogen addition altered the network complexity. It may be that the nitrogen application treatment selectively impacted the reproduction and growth of soil microorganisms, causing changes in the interactions between microorganisms and thereby affecting the complexity of the network structure. Among these, positive links dominated in the three treatments, implying that mutual cooperation more than competitive exclusion played an important role in the microbial community. The LN reduced the interactions between species, either by mutual cooperation or competitive exclusion. The amount of NH_4_^+^ may be insufficient to significantly alleviate the limit of soil nitrogen use by the soil bacteria. Consistent with Sha et al. [49], who found that nitrogen addition reduced positive mutualism and depressed coprological interactions between soil microbial species, Yu et al. 2019 showed that continued urea addition reduces the redundancy of species interactions. Under conditions of limited N content, most of the nitrogen applied in soil may be absorbed by vegetation rather than by soil bacteria [49,87] because vegetation may be more competitive than soil bacteria. The HN increased mutual cooperation between species but decreased interspecies competition. The application of NH_4_^+^ provided an enriched nitrogen source for bacteria, which reduced competition for limited resources and promoted cooperation among species. Consistent with Li et al. [13], who investigated the effect of nitrogen fertilization on the network structure of soil microorganisms, this study found that excess N addition enriched a number of mutualistic microbes and increased the percentage of positive links. However, the input of NH_4_^+^ decreased the number of modules and negative links in the bacterial network, indicating that nitrogen addition reduced the stability of the soil bacterial community [88,89]. Exogenous nitrogen inputs may cluster species that are sensitive to nitrogen fertilization and thereby have an impact on the bacterial community [13]. Similar results were obtained by Yao et al. [58], who found that high nitrogen addition reduced the stability of the microbial systems. The decrease in stability may be attributed to a loss of diversity and changes in community composition caused by nitrogen enrichment effects [90].

## 5. Conclusions

Our study investigated the effects of more than five years ammonium addition on soil bacterial diversity and composition in the *D. angutifolia* Wetland of Sanjiang Plain. High nitrogen addition significantly reduced the heterogeneity of the soil bacterial community and had no significant effect on the richness, whereas low nitrogen addition did not alter the soil bacterial diversity. NH_4_^+^ addition changed the bacterial composition and the community network complexity and stability in the *D. angutifolia* Wetland of Sanjiang Plain. SMC, NO_3_^−^, and pH were key soil parameters driving the composition of soil bacterial community structures. These results provide a theoretical basis for studying the impacts of future climate change on the diversity and functional stability of wetland ecosystems.

## Figures and Tables

**Table 1:** Physicochemical characteristics of the soil samples for the different N treatments.

Treatments	SMC	pH	NO_3_ (mg/kg)	NH_4_^+^ (mg/kg)	DOC (mg/kg)	DON (mg/kg)	TN (%)	SOC (%)
CK	0.43 ± 0.06 b	4.41 ± 0.43 a	23.83 ± 6.34 b	21.15 ± 3.45 a	3897.88 ± 625.37 b	299.74 ± 130.71 b	0.57 ± 0.23 b	5.34 ± 2.23 a
LN	0.48 ± 0.01 ab	4.19 ± 0.06 ab	23.27 ± 3.49 b	5.62 ± 0.63 b	2571.62 ± 294.54 c	520.08 ± 166.01 ab	0.68 ± 0.05 ab	4.53 ± 0.42 a
HN	0.58 ± 0.05 a	4.02 ± 0.11 b	40.60 ± 6.17 a	4.72 ± 0.06 b	5004.81 ± 331.11 a	719.68 ± 156.87 a	1.18 ± 0.29 a	6.86 ± 2.00 a

Note: Each treatment was performed in triplicate. The data are expressed as the mean ± standard deviation; lowercase letters indicate significant differences (*p* < 0.05). CK: control; LN: low ammonium; HN: high ammonium. SMC: soil moisture content; DOC: dissolved organic carbon; DON: dissolved organic nitrogen; TN: total nitrogen; SOC: soil organic carbon.

**Table 2:** Parameters of bacterial network under different N addition levels.

Treatment	OTU Numbers	Total Links	Positive Link	Negative Link	Average Degree	Average Clustering Coefficient	Average Geodesic Distance	Network Density	Modularity (No. of Modules)
CK	465	11,902	6637	5265	25.6	0.784	11.723	0.055	0.812 (11)
LN	464	10,922	5809	5113	23.5	0.791	11.604	0.051	0.824 (10)
HN	458	12,542	7329	5213	27.4	0.812	11.409	0.060	0.779 (9)

CK: control; LN: low ammonium; HN: high ammonium.

## Data Availability

All raw sequences have been deposited into an NCBI Sequence Read Archive with the BioProject accession: PRJNA880534.

## Supplementary Materials

The following supporting information can be downloaded at: https://www.mdpi.com/article/10.3390/microorganisms11082033/s1.
