# Supplementary material for: High Ammonium Addition Changes the Diversity and Structure of Bacterial Communities in Temperate Wetland Soils of Northeastern China
Source: Microorganisms. 2023 Aug 8;11(8):2033. doi: 10.3390/microorganisms11082033 (PMC10459003; doi:10.3390/microorganisms11082033)
Supplement: Supplementary file 1 [file microorganisms-11-02033-s001.zip › microorganisms-2506961-supplementary.pdf]

Table S1. PERMANOVA (ADONIS) analysis of bacteria comparing the three N addition treatments.

	R^2	*P*
CK versus LN versus HN	0.48	0.008
CK versus LN	0.24	0.2
CK versus HN	0.45	0.1
LN versus HN	0.48	0.1

CK: control; LN: low ammonium; HN: high ammonium.

Table S2. Permutational analysis of multivariate dispersions (PERMDISP) analysis of bacteria comparing the three N addition treatments.

Centroid of CK	Centroid of LN	Centroid of HN	F	*P*
0.2344	0.1969	0.211	1.5622	0.312
0.2344	——	0.211	1.0679	0.4014
0.2344	0.1969	——	2.1614	0.4014
——	0.1969	0.211	0.8061	0.4014

CK: control; LN: low ammonium; HN: high ammonium.

Table S3. Significance of the soil physicochemical properties in explaining the bacterial community structure obtained from the RDA results.

	R^2	*P*
SMC	0.820	<0.01
pH	0.783	<0.05
NO_3^-	0.787	<0.01
NH_4^+	0.403	>0.05
DOC	0.639	<0.05
DON	0.591	>0.05
TN	0.767	<0.05
SOC	0.373	>0.05

CK: control; LN: low ammonium; HN: high ammonium. SMC: soil moisture contents; DOC: dissolved organic carbon; DON: dissolved organic nitrogen; TN: total nitrogen; SOC: soil organic carbon.

Table S4. Pearson correlation of soil variables with alpha-diversity indices

	SMC	pH	NO_3^-	NH_4^+	DOC	DON	TN	SOC
Chao index	-0.38	0.22	-0.33	0.28	-0.45	-0.17	-0.45	-0.22
Shannon index	-0.76*	0.63	-0.67*	0.54	-0.62	-0.59	-0.76*	-0.47
Simpson index	0.69*	-0.52	0.63	-0.38	0.68*	0.49	0.71*	0.49

CK: control; LN: low ammonium; HN: high ammonium. SMC: soil moisture contents; DOC: dissolved organic carbon; DON: dissolved organic nitrogen; TN: total nitrogen; SOC: soil organic carbon. *represents significant spearman correlation at 0.05 level.

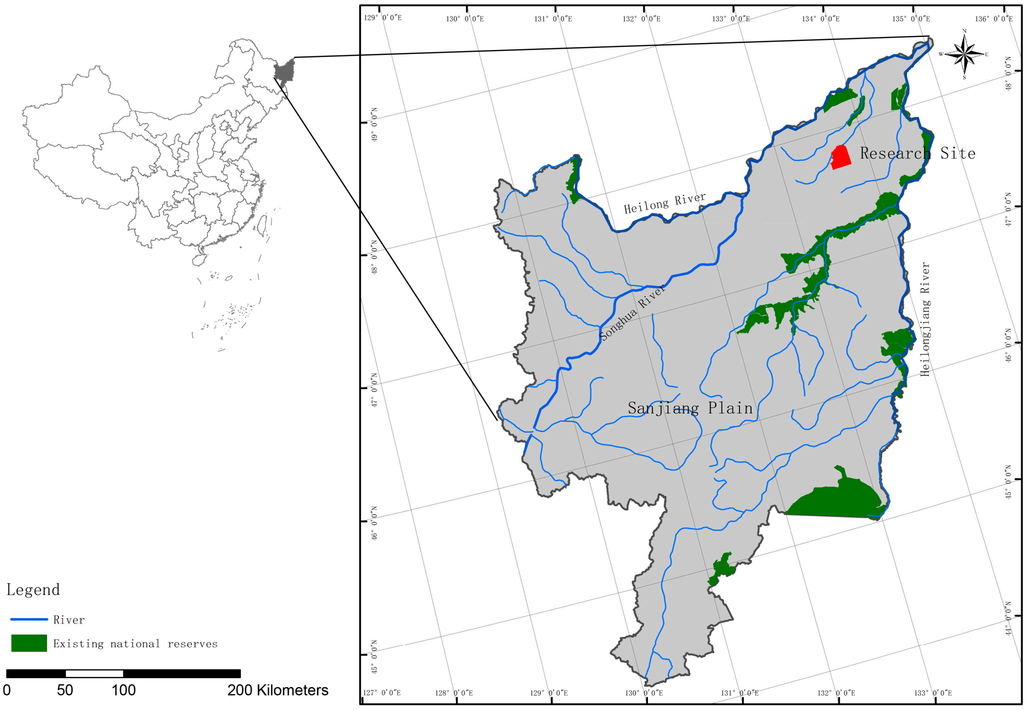

Figure S1. Honghe nature reserve in Sanjiang Plain.

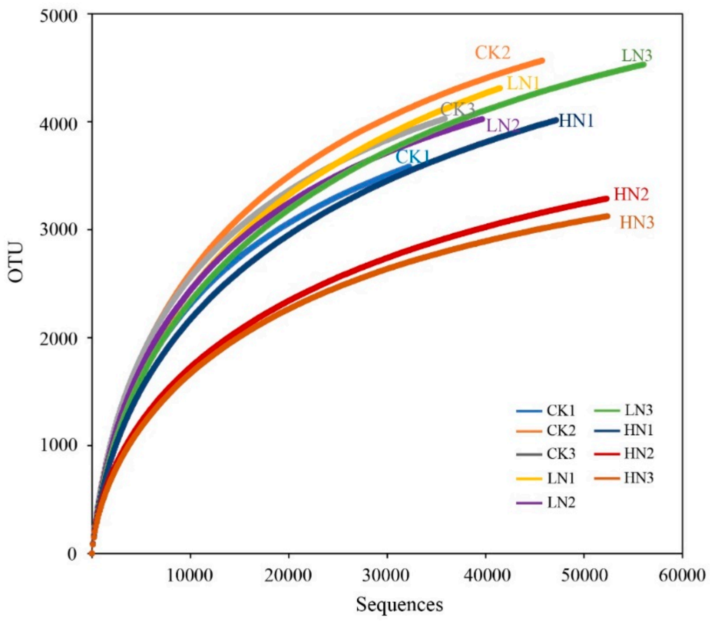

Figure S2. Rarefaction curves of soil bacteria in the different nitrogen treatment. CK: control; LN: low nitrogen; HN: high nitrogen.

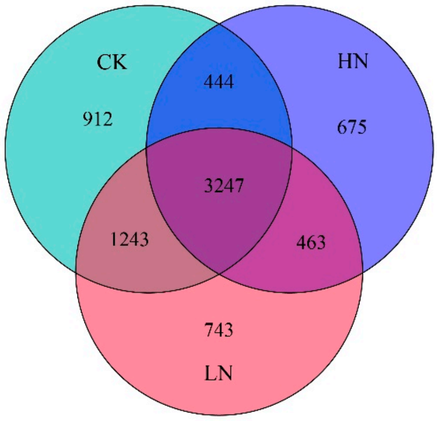

Figure S3. Venn diagram showing the numbers of shared and exclusive OTUs in different nitrogen treatments. CK: control; LN: low ammonium; HN: high ammonium.

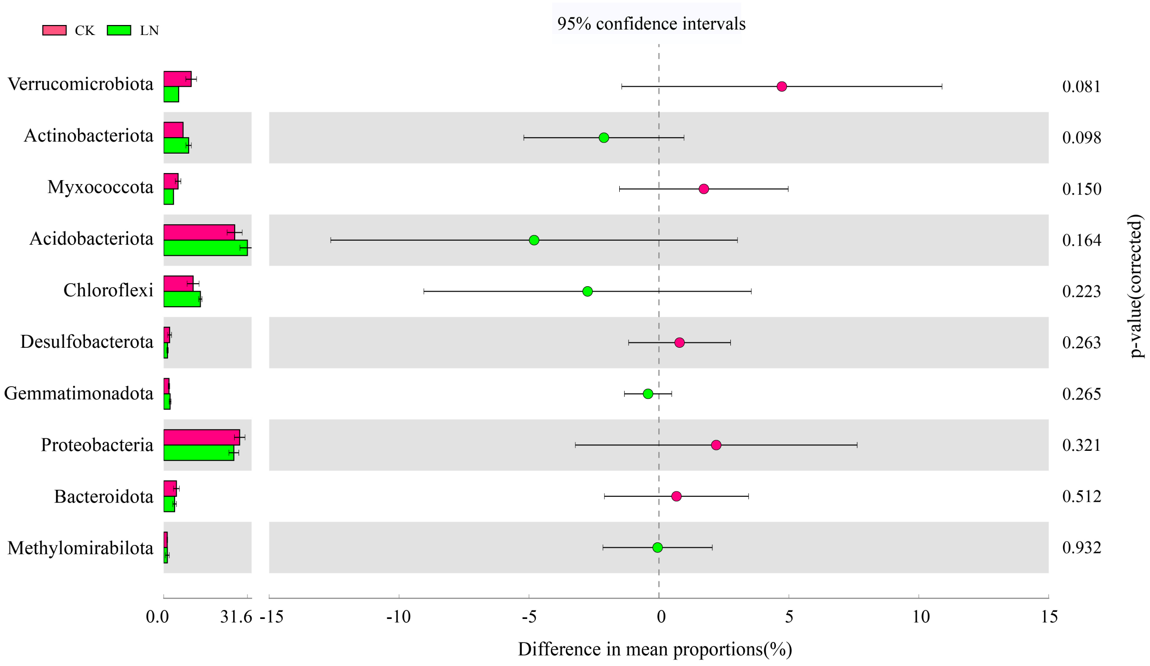

Figure S4. Relative abundance of bacterial phyla (relative abundance top 10) under different N addition levels (CK versus LN). Values in the bar plot are expressed as mean ± standard error. The colored circles represent the 95% confidence intervals. * indicated a significant difference at 0.5 level. CK: control; LN: low ammonium.

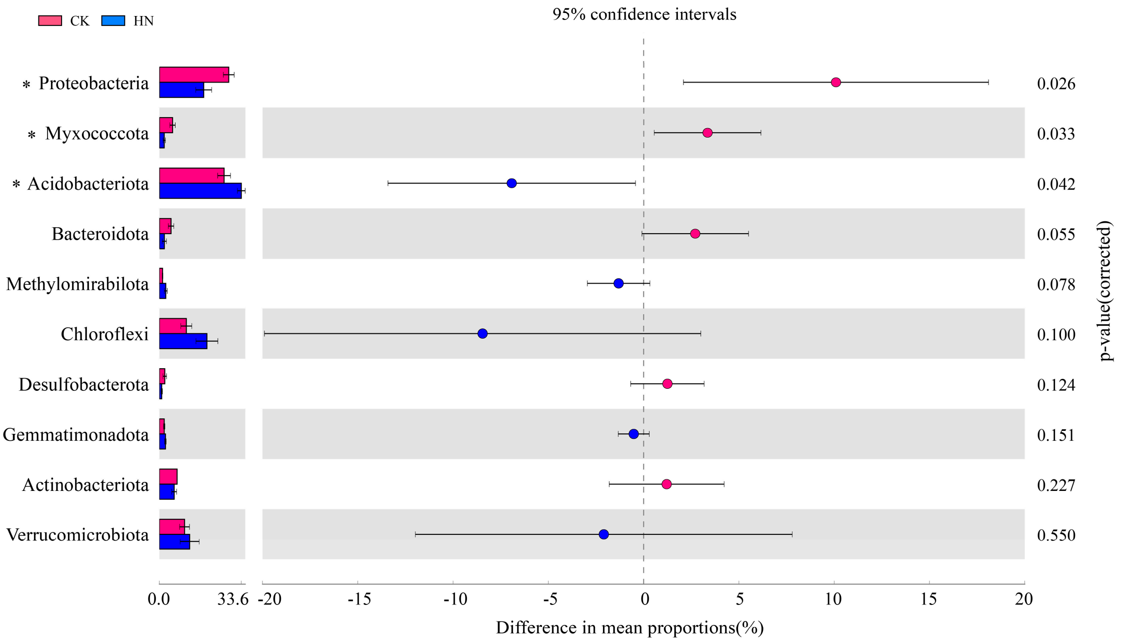

Figure S5. Relative abundance of bacterial phyla (relative abundance top 10) under different N addition levels (CK versus HN). Values in the bar plot are expressed as mean ± standard error. The colored circles

represent the 95% confidence intervals. * indicated a significant difference at 0.5 level. CK: control; HN: high ammonium.

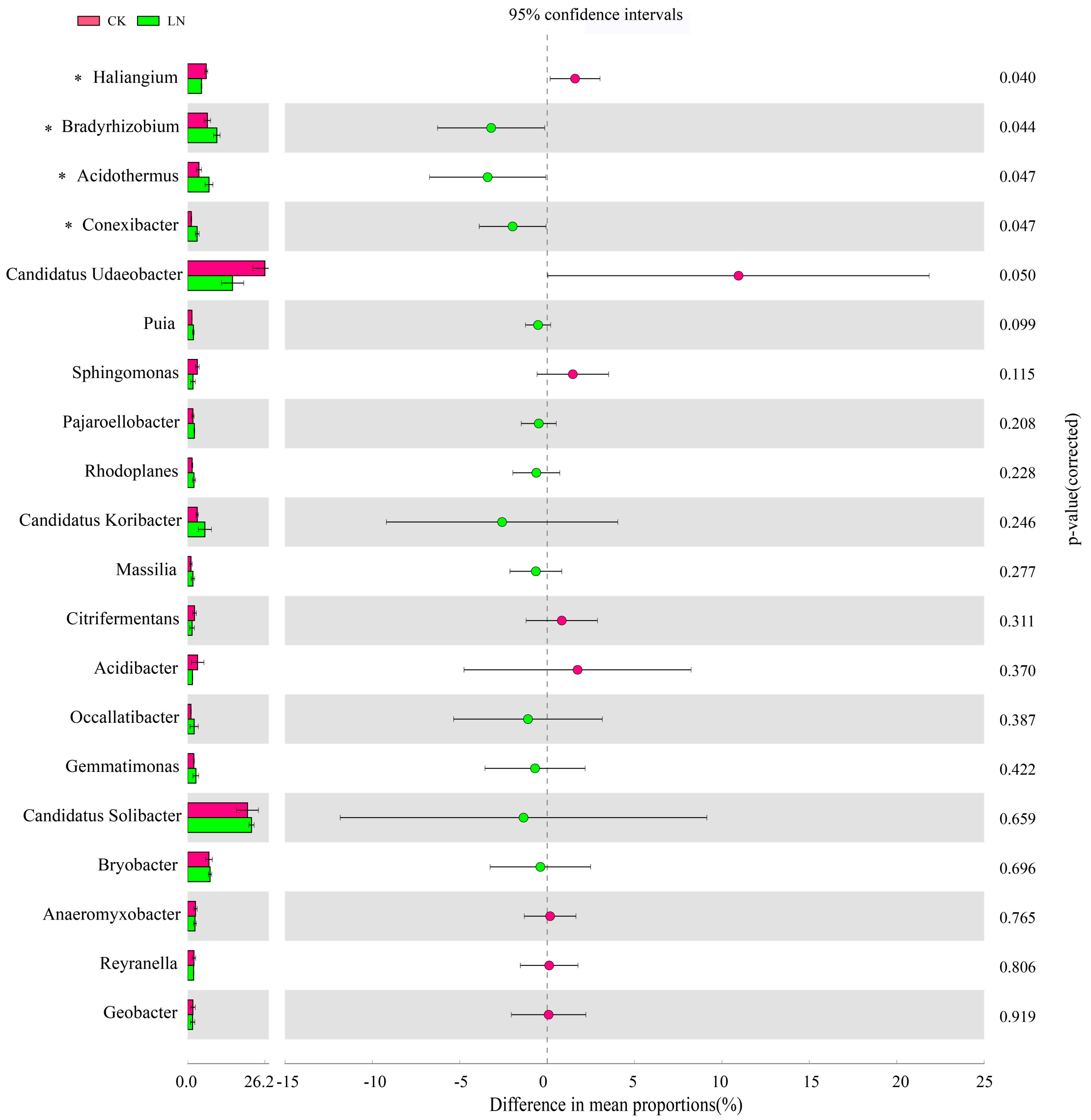

Figure S6. Relative abundance of bacterial genera (top 20 genera were shown) under different N addition levels (CK versus LN). Values in the bar plot are expressed as mean ± standard error. The colored circles represent the 95% confidence intervals. * indicated a significant difference at 0.5 level. CK: control; LN: low ammonium.

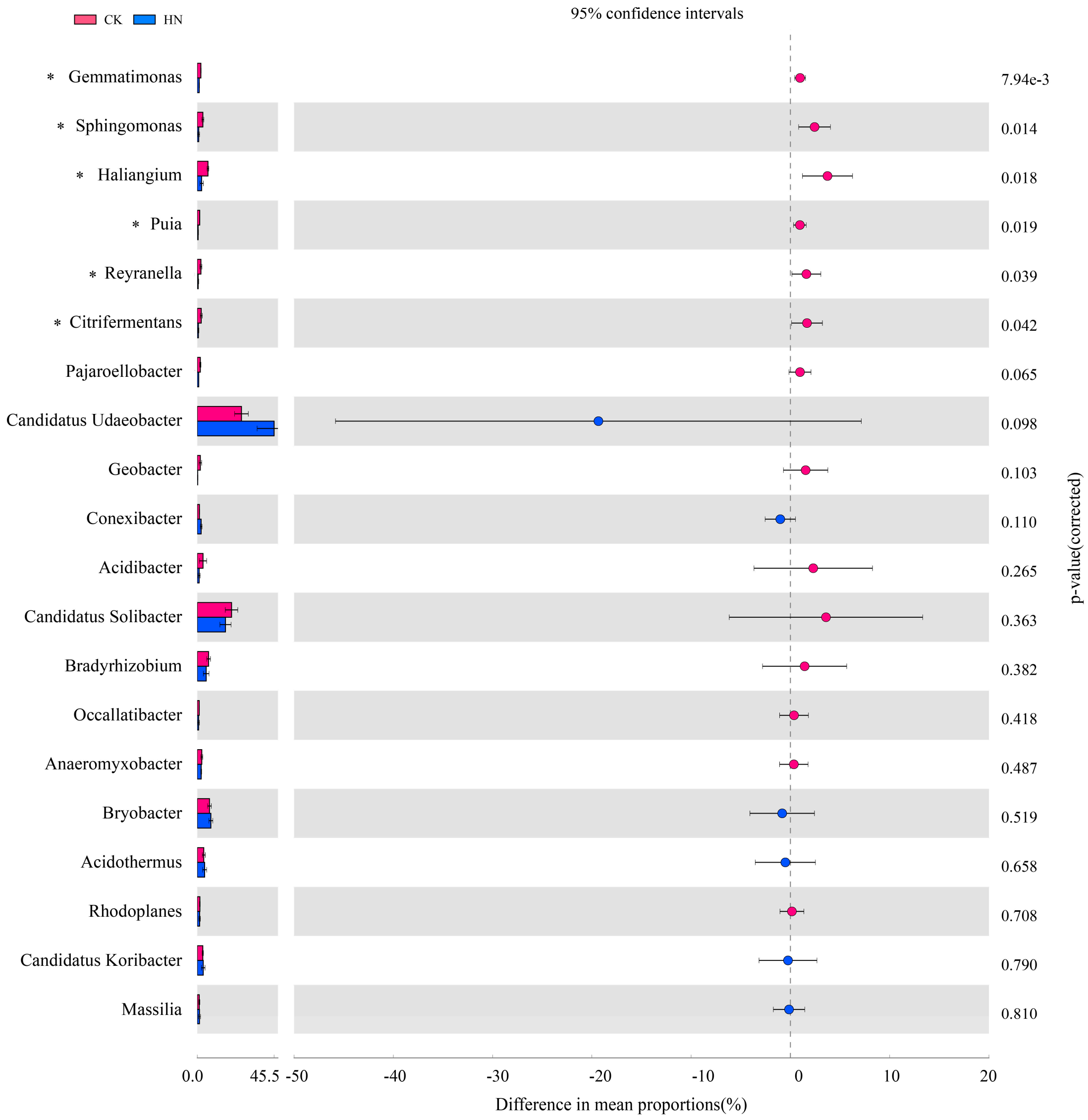

Figure S7. Relative abundance of bacterial genera (top 20 genera were shown) under different N addition levels (CK versus HN). Values in the bar plot are expressed as mean ± standard error. The colored circles represent the 95% confidence intervals. * indicated a significant difference at 0.5 level. CK: control; HN: high ammonium.
